# The potential of Real-Time rendered-volume 3D Imaging in immersive virtual-reality (VR) for surgical planning in infants with congenital thoracic malformation (CTM)

**DOI:** 10.1016/j.csbj.2025.11.005

**Published:** 2025-11-05

**Authors:** D. Zalepugas, J. Buermann, S. Senkel, N. Schmidt, AM Ziegler, R. Kurz, J. Schmidt, P. Feodorovici, J. Arensmeyer

**Affiliations:** a Department of Thoracic Surgery, University Hospital Bonn, Bonn, Germany; b Department of Thoracic Surgery, Helios Hospital Bonn/Rhein-Sieg, Bonn, Germany; c Department of General, Visceral-, Vascular, and Transplant Surgery, Pediatric Surgery Department, University Hospital Bonn, Bonn, Germany

**Keywords:** digital twin, congenital thoracic malformation, virtual reality, precision medicine, surgical planning, smart hospital

## Abstract

**Background:**

CTM are difficult to evaluate preoperatively due to the limitations of low-dose computed tomography imaging in infants. Digital twin technologies, such as immersive VR, offer interactive, patient-specific simulations that replicate anatomical and pathological features in three dimensions. This study investigates the role of VR-based 3D imaging as a digital twin for precision surgical planning in infants with CTM.

**Methods:**

Fourteen infants who underwent surgical resection for suspected CTM between 09/2020 and 02/2023 were retrospectively analyzed. Pseudonymized CT scans were reconstructed into interactive VR models, serving as digital anatomical twins. Eight surgeons assessed each case using both conventional 2D CT and VR-based reconstructions, separated by a three-month washout period. Outcomes included diagnostic accuracy, and evaluation times.

**Results:**

Both VR and conventional imaging achieved 100 % sensitivity in pathology detection. VR enhanced spatial understanding, identifying multilobar involvement (29 % vs. 21 %) and mediastinal shift (36 % vs. 29 %) more frequently. Surgical planning differed significantly, with VR prompting more lobectomy recommendations (57 % vs. 50 %, p = 0.046). VR required longer evaluation times (203.2 ± 117.4 s vs. 124.1 ± 69.7 s, p < 0.001) but demonstrated higher prediction accuracy for the surgical procedure (95 % vs. 84 %).

**Conclusion:**

Patient-specific VR reconstructions act as digital twins, enhancing surgical planning precision and providing a personalized anatomical roadmap for CTM management. While time demands remain a limitation, VR as a digital twin aligns with precision medicine and smart hospital paradigms. It also supports less experienced surgeons and fosters interdisciplinary collaboration. Larger multicenter studies should explore its integration into perioperative decision-making and training.

## Highlights

• VR-based 3D reconstructions act as anatomical health digital twins for congenital thoracic malformations.
• Immersive VR improved surgeons’ spatial understanding and diagnostic accuracy despite low resolution in infants.
• Surgical assessment was more accurate after VR review, especially toward lobectomy.
• Less experienced surgeons benefited most from the VR-based digital twin approach.
• VR integration supports precision medicine and smart hospital surgical planning.

## Graphical abstract

**Figure:**
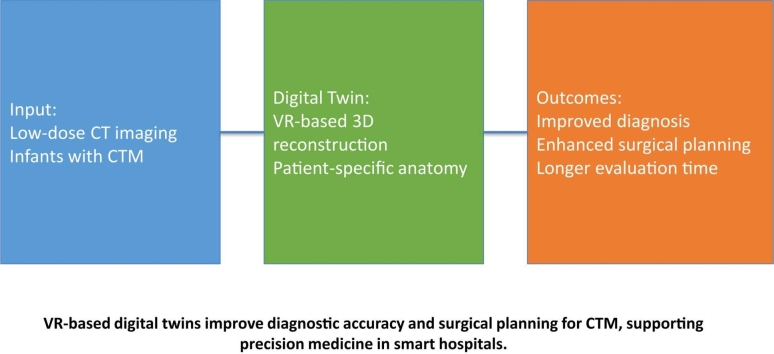

**Highlight box**

**Key findings**

• VR-based reconstructions function as health digital twins for CTM, offering personalized, patient-specific anatomical simulations.
• VR can improve spatial mapping of complex anatomy and could significantly influence surgical planning.
• Less experienced surgeons particularly benefited from the digital twin approach, compensating for limited clinical exposure.

**What is known and what is new?**

• Conventional CT imaging is the standard for CTM evaluation but is limited by poor spatial resolution in infants.
• This study introduces VR as a clinical digital twin, indicating feasibility in surgical planning and highlighting its role in precision and personalized pediatric thoracic surgery.

**What is the implication, and what should change now?**

• VR-based digital twin technologies should be integrated into smart hospital workflows to support interdisciplinary patient-specific surgical planning, training, and patient-specific precision medicine.
• Wider adoption and validation of VR-based digital twins could redefine surgical preparedness, particularly in complex congenital conditions.

## 1 Introduction

### 1.1 Background

The term cngenital thoracic malformation (CTM) covers a broad range of lung anomalies, including congenital pulmonary airway malformation (CPAM), previously known as congenital cystic adenomatous malformation; intra- and extra-lobar pulmonary sequestration (PS); bronchogenic cysts; bronchial atresia; congenital lobar hyperinflation; congenital large hyperlucent lobe (CLHL); and vascular anomalies. [1], [2]

Infants with respiratory insufficiency, a mediastinal shift present on postnatal imaging, a high CPAM volume ratio (CVR) or a large lesion during gestation are more likely to require surgery. [1] Arguments for recommending an early active surgical approach for asymptomatic infants include the possibility of developing respiratory symptoms, such as a persistent cough or recurrent airway infections. The latter may cause lung and pleural alterations, which can increase the risk of complications during or after surgery. Furthermore, early resection could facilitate optimal lung growth of the rest of the lobe. Another frequently mentioned argument is the possible relationship between CPAM and malignant degeneration, like bronchioalveolar carcinoma and pleuropulmonary blastoma. [1], [3]

These malformations are generally diagnosed by antenatal and postnatal ultrasound imaging, as well as targeted magnetic resonance tomography (MRI). A low-dose computerized tomography (CT) scan is used for the preoperative evaluation to minimize radiation exposure to the patient during this sensitive stage of development. Furthermore, high-resolution imaging is unavailable due to the infant’s small body volume. Recent research by Style et al. highlights the advantages of a preoperative CT scan versus an MRI. Postnatal CT scans had the highest diagnostic specificity and the best prediction of postoperative CTM lesion histology. [4], [5]

### 1.2 Rationale and knowledge gap

Despite having sufficient experience with contrast-enhanced chest CT, many CTMs may be subtle or invisible on initial chest radiographs. There is significant overlap in imaging characteristics among conditions such as CPAM and bronchopulmonary sequestration (BPS), which complicates differentiation. Current studies have identified the need to improve the clarity of CT imaging findings. [6], [7]

Three-dimensional (3D) reconstruction has the potential to improve the accuracy of patient-specific surgical planning. A recent publication by Bakhuis et al. demonstrated the significant impact of 3D reconstructed imaging in a virtual reality environment on decision-making for lung segmentectomies. Recent technological progress has increased the availability of head-mounted display (HMD) hardware and comprehensive software. This technology now enables collaborative VR sessions to view, edit, and discuss volume-rendered CT imaging. [8], [9] This technology provides a more realistic representation of the preoperative imaging for surgeons, enhancing their understanding of surgical targets and improving their spatial comprehension and better identification of anatomical structures. [10], [11], [12], [13]

The surgical indication is decided through interdisciplinary consensus with a neonatologist, a child radiologist, and a child intensive care physician. In practice, the surgeon carries the final responsibility for the indication and intraoperative decisions. Therefore, we decided to perform a separate evaluation for surgeons. Recent studies have already evaluated the role of viewer age in VR usage. [14], [15] On the other hand, a surgeon's experience is undoubtedly an important independent factor in diagnostics and decision-making. [16]

### 1.3 Objective

We describe a novel method for surgical planning using live volume-rendered 3D imaging in a low-dose CT setting using a virtual reality HMD. Our primary hypothesis is that 3D visualization has the potential to improve the surgeon’s assessment through combining multislice CT scans into one 3D model, allowing for simultaneous visualization of multiple anatomical structures in their native spatial relationships. This may help to better identify the pathology and enhance the preoperative surgical plan. Our secondary hypothesis is that less experienced surgeons may have an advantage with VR due to generational affinity. This could compensate for their lack of clinical experience.

## 2 Materials and methods

### 2.1 Patient inclusion and exclusion

A total of 14 infants who underwent surgical resection for suspected CTM and were later histologically confirmed at the University Hospital Bonn, a tertiary care center in Germany, between August 2020 and February 2023, were enrolled in this study. The exclusion criterion was the presence of other potentially misleading synchronous pathologies such as diaphragmatic herniation or hemothorax.

### 2.2 Participants

Four resident surgeons and four senior surgeons were involved in the study. The resident surgeons had an average of 18 months of respective clinical experience and the senior surgeons an average of 7 years. None of them were involved in the initial diagnostics of the given cases.

### 2.3 Study design

**Fig. 1:**
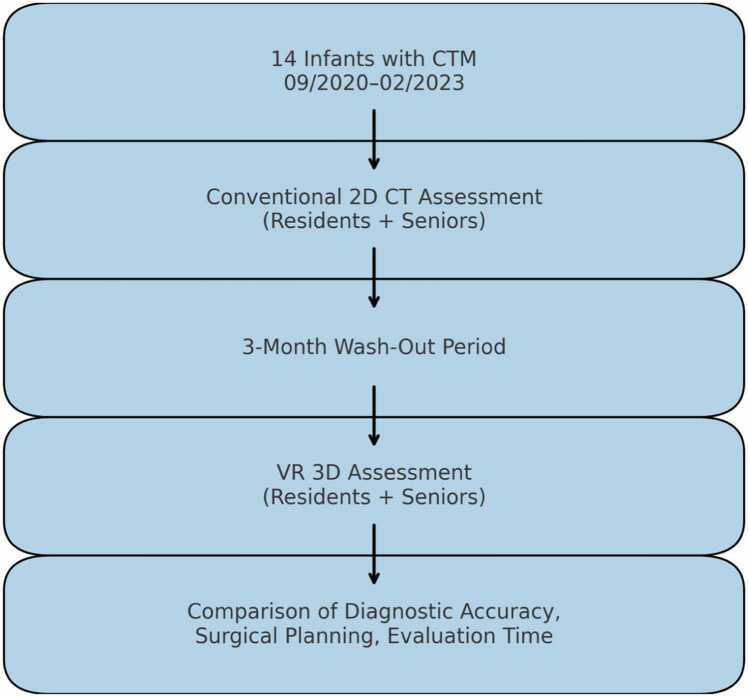
Study design.

For means of our study, we performed individual retrospective assessments of the pseudonymized CT scans in both conventional 2D view and VR displayed 3D rendered CT scan data. The Study design is depicted in Fig. 1. The participants were first asked to evaluate the CT scans in the conventional 2D view. After a washout period, VR-based planning was used for the same CT scans. Case order within each evaluation block was randomized to minimize recall bias. The assessments were correlated with findings from the histopathological and surgical reports.

### 2.4 Conventional CT evaluation setup

The conventional CT scan evaluation was performed on the hospital’s standard computer equipment, consisting of a HP Elitedesk (HP Inc. Palo Alto, CA, USA) with an Intel Pentium I7 Processor (Intel Corp., Santa Clara, CA, USA) and 16 GB of Random Access Memory, with a 24-inch HP (HP Inc., Palo Alto, CA, USA) Full High Definition screen. The CT scans were loaded and displayed in axial slices using Xero Viewer (Agfa Healthcare, Mortsel, Belgium). The user interaction was enabled by the computer’s keyboard and mouse.

### 2.5 VR evaluation setup

The VR evaluation was performed in a dedicated room with a freely accessible area of approximately 20 square meters. The Meta Quest 3 was used as the HMD. The image data was processed and rendered on an on-premises server infrastructure located in the secured hospital network and equipped with multiple NVIDIA A40 GPUs (Nvidia Corp., Santa Clara, CA, USA), then streamed to the HMD via the hospital's own Wi-Fi infrastructure. Medical Imaging XR (Medicalholodeck AG, Zurich, Switzerland) software was used to visualize the low-dose CT image data in the VR environment. The patients' CT data were imported as digital imaging and communications in medicine (DICOM) files directly from the hospital's own picture archiving and communication system (PACS) server into the Medical Imaging XR software, while importing the data from the PACS the Medical Imaging XR software automatically pseudonymized the data. As all data were handled within the hospital’s secured network infrastructure; no patient-identifiable information left institutional servers. Participants interacted with the imaging using the HMD’s controllers. Due to the HMD's inside-out tracking, no additional sensors were required. The pseudonymized data was then selected by the user to be displayed in the VR-environment. “Color grading“ was performed using sliders in the software‘s user interface. Specific colors were assigned to certain Hounsfield ranges to create a color representation of the CT image data. A template created by our working group was provided to the participants as a baseline. It primarily highlights the contrast between areas filled with air and areas with higher parenchymal density. Although collaborative viewing and processing of the CT image data is possible with multiple instances, it was not used here due to the single-user study design. Real-time streaming achieved a minimum framrate of 60 frames per second with latencies below 50 ms, ensuring smooth interaction.

### 2.6 Data collection

The original clinical surgical planning was performed using conventional 2D CT grayscale imaging on a computer monitor. For the VR-based assessment an optimal color grading preset for the 3D model was provided for the participants. Windowing, thresholding, and color grading parameters were standardized across all cases and participants using the preset function of the vendor’s windowing and color grading tool.

**Fig. 2:**
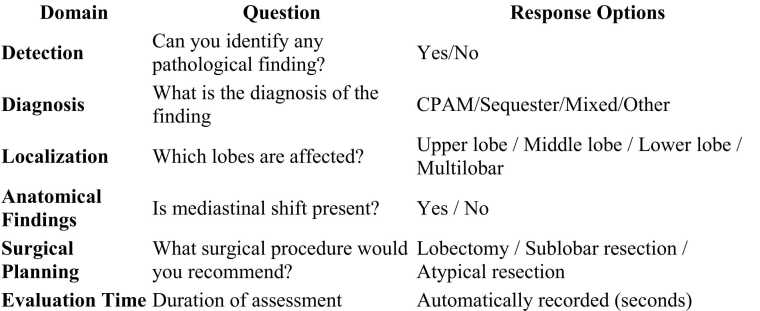
Questionnaire.

All the participants were acquainted with interpreting 2D CT scans according to their experience level. Every participant was given a VR system introduction, practical instruction and taught how to adjust color grading. The introduction and getting used to controls were limited to 45 min. The surgeon was then instructed on the evaluation process and made acquainted with the survey criteria. The surveyor registered the processing time. A subsequent collection of assessments using a questionnaire was performed. The questionnaire is shown in the ‘’results’’ section Fig. 2.

In the second phase of our study, we used a visual memory washout period. According to the current literature, an adequate washout period after a median exposure time of under 5 min per case is at least 2 months. [17] We implemented a 3-month washout period after which the CT scans were pseudonymized again and surgeons assessed them using the other method.

The primary outcome was the incidence of changes in surgical plan and predicted pathology. We compared plans made with VR-based planning to those made with conventional 2D CT alone. The secondary study outcome was the observed processing time difference in dependence on surgeon experience.

This study was approved by the Medical Ethical Committee of the University of Bonn (No. 2024–316-BO).

### 2.7 Statistical analysis

The questionnaire data were converted into categorical variables reported as counts and percentages.

The Wilcoxon signed-rank test was used to compare two related samples to see if their population mean ranks differ. Continuous variables are reported as medians and ranges or means where appropriate and were analyzed using two-tailed Student’s *t*-tests or Mann-Whitney U tests where appropriate. Categorical variables were compared using a McNemar’s test or Chi-squared test where appropriate. Statistical significance was deemed at p < 0.05.

Effect sizes (Cohen’s d or Cramer’s V) and 95 % confidence intervals were reported where appropriate. No formal correction for multiple comparisons was applied given the exploratory nature of this feasibility study; this limitation is acknowledged.

Cohen’s kappa coefficient was used to measure the agreement between the two evaluation groups, in order to differentiate how much better the raters agree than random guessing. Statistical analysis was performed using Microsoft Excel and MedCalc version 23.1.3 statistical software.

## 3 Results

**Fig. 3:**
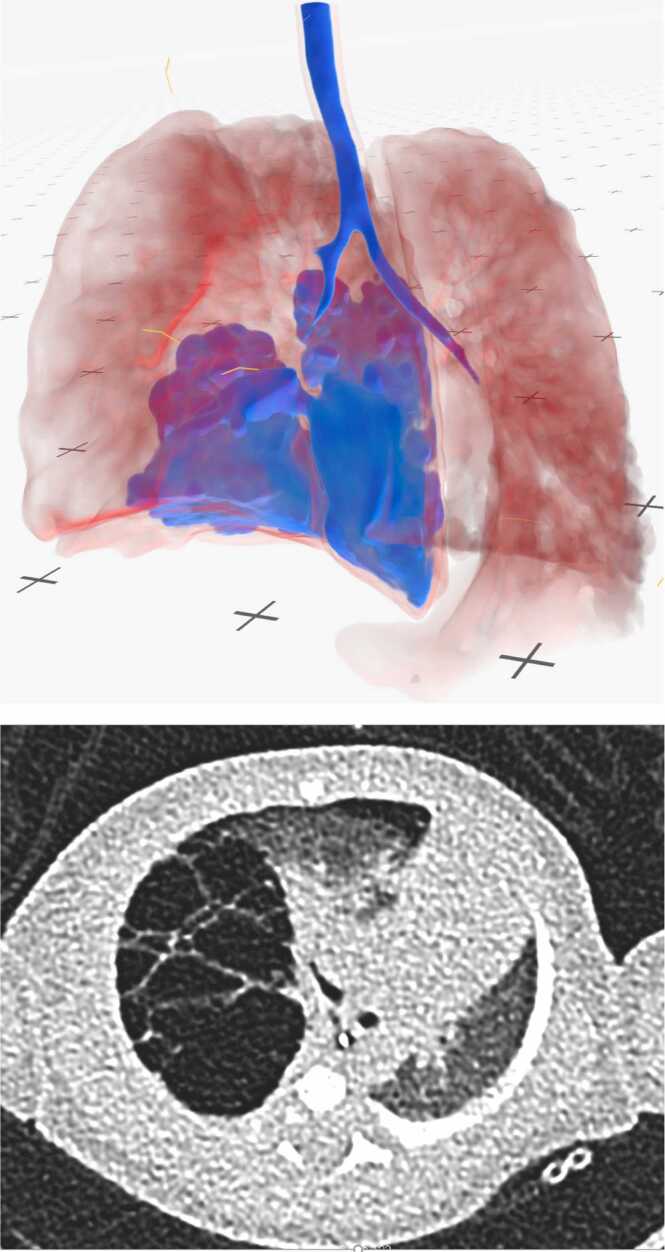
Comparison of visualization quality (VR vs Conventional).

Eight participants evaluated 14 identical cases using both the VR-based analysis and the conventional 2D image evaluation (Fig. 3), resulting in a total of 112 paired and 224 total assessments. The pathological diagnosis, radiological reports, and intraoperative surgical findings served as the reference standards, depending on the parameter assessed.

The 14 cases included 10 male and 4 female patients (Table 1). The mean patient’s age at the date of imaging was 9.36 days with a standard deviation (SD) of 28.15, with a range of 0–103 days and a median of 1.5 days. The mean interval between imaging and subsequent surgery was 3.36 days (SD 1.65), ranging from 1 to 7 days, with a median of 3.5 days. The mean age at surgery was 12.71 days (SD 28.77), with a minimum of 2 days, a maximum of 109 days, and a median of 5.5 days.

**Table 1:** Patient cohort characteristics (n = 14).

**Parameter**	**Value**
Sex (male/female)	10 / 4
Mean age at imaging (days) ± SD (range)	9.4 ± 28.2 (0–103)
Median age at imaging (days)	1.5
Mean interval imaging → surgery (days) ± SD (range)	3.4 ± 1.7 [1], [2], [3], [4], [5], [6], [7]
Median interval imaging → surgery (days)	3.5
Mean age at surgery (days) ± SD (range)	12.7 ± 28.8 (2–109)
Median age at surgery (days)	5.5

**Table 2:** Diagnostic and surgical planning outcomes: VR vs conventional assessment.

Parameter	N paired	3D-VR correct	2D conventional correct	Agreement	McNemar exact p
Pathology detection	112	112/112 (100.0 %)	112/112 (100.0 %)	100.0 %	1.000
Diagnosis (reference: pathology)	112	92/112 (82.1 %)	82/112 (73.2 %)	85.7 %	0.021
Diagnosis (reference: radiology)	96	93/96 (96.9 %)	87/96 (90.6 %)	89.6 %	0.109
Localization (reference: surgery)	112	77/112 (68.8 %)	80/112 (71.4 %)	77.7 %	0.690
Localization (reference: radiology)	112	85/112 (75.9 %)	87/112 (77.7 %)	69.6 %	0.864
Mediastinal shift	56	36/56 (64.3 %)	36/56 (64.3 %)	75.0 %	1.000
Procedure identification	112	60/112 (53.6 %)	62/112 (55.4 %)	71.4 %	0.860

For pathology detection, both methods achieved perfect accuracy (112/112, 100 %), with complete agreement and no discordant pairs (McNemar’s test: p = 1.000).

For diagnosis referenced against pathology, the VR-based method outperformed the conventional approach (82.1 % vs. 73.2 %). This difference was statistically significant (McNemar’s exact test: p = 0.021), with 13 cases correctly identified by VR but missed by the conventional method, compared to 3 cases vice versa.

For diagnosis referenced against radiology, accuracy was higher with VR (96.9 %) than with the conventional method (90.6 %), indicating a no statistically significant difference in favor of VR (p = 0.109).

For lesion localization referenced against surgery**,** accuracy was comparable between VR and conventional testing (68.8 % vs. 71.4 %), with no significant difference (p = 0.690). Similarly, for localization referenced against radiology, performance was nearly identical (75.9 % vs. 77.7 %; p = 0.864).

For mediastinal shift**,** both modalities achieved identical accuracy (64.3 %), with symmetric discordant pairs (b = c = 7) and no significant difference (p = 1.000).

For procedure identification**,** performance was slightly higher with VR (80.4 % vs. 75.0 %), but this difference did not reach statistical significance (p = 0.424).

**Fig. 4:**
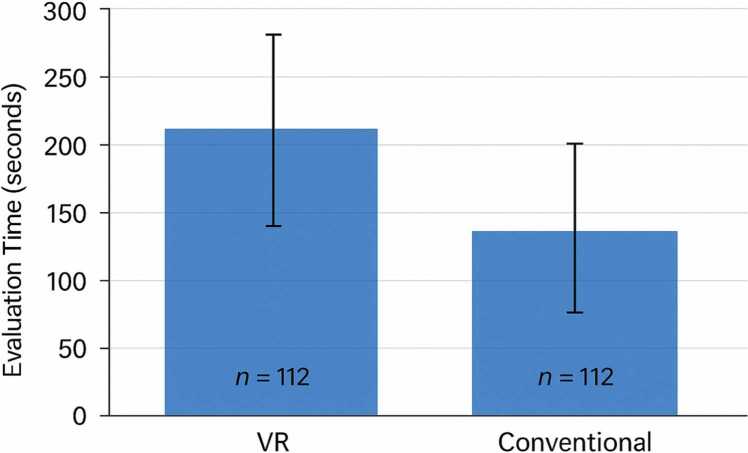
Comparison of evaluation times (VR vs Conventional).

Evaluation times for 14 pediatric CPAM cases differed significantly between virtual reality and conventional CT-based assessments (Fig. 4). VR assessments were consistently slower, with a mean duration of 203.2 ± 117.4 s compared to 124.1 ± 69.7 s for conventional evaluations (paired *t*-test, p < 0.001). VR times ranged from 55 to 683 s, while conventional times spanned 36–260 s. Median times were 187 s for VR and 108.5 s for conventional assessments, with broader variability in the VR group.

Both datasets followed normal distributions (Shapiro-Wilk test, p > 0.1). The mean difference between methods was 53.2–105.0 s (95 % CI), corresponding to a large effect size (Cohen’s d = 1.74). VR times correlated with multilobar involvement (ρ = 0.61, p = 0.02) and decreased with case order (ρ = –0.54, p = 0.04), indicating potential efficiency gains over time. Conventional times showed no statistically significant difference with these factors.

Inter-rater agreement differed across parameters and was generally equal or higher in VR compared with conventional assessment. For diagnosis referenced against pathology, VR demonstrated substantial agreement, whereas the conventional method reached only moderate agreement. Agreement levels for localization tasks were moderate in both methods, and mediastinal shift showed fair to moderate agreement without notable differences between modalities.

**Fig. 5:**
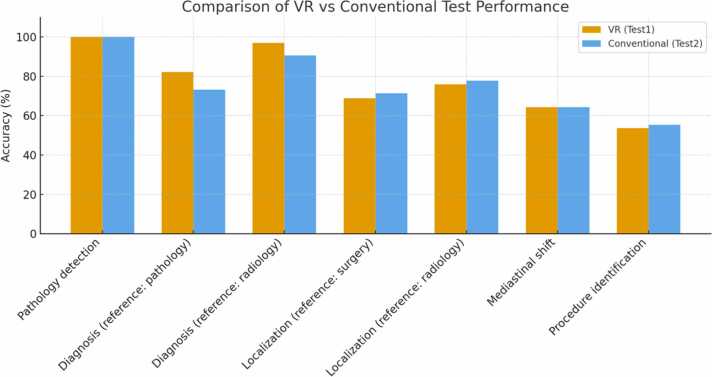
Diagnostic and Surgical Planning accuracy.

Overall, the VR-based method demonstrated comparable or superior accuracy across all parameters, with a statistically significant advantage observed for diagnosis referenced against pathology. In contrast, VR assessments required significantly more time than conventional evaluations, with broader variability and two markedly prolonged cases. Notably, VR evaluation times showed a negative correlation with case order, suggesting improved efficiency over time, while conventional assessments displayed no statistically significant difference. These findings are illustrated in Fig. 5, which summarizes diagnostic performance and evaluation times across methods.

## Discussion

This study demonstrates that immersive VR reconstructions can serve as digital anatomical twins for infants with CTM. These models are derived from low-dose CT scans. By enabling interactive exploration of patient-specific thoracic anatomy, VR supports more precise spatial understanding compared to conventional two-dimensional imaging.

### 3.1 Diagnostic and anatomical assessment

VR and conventional CT imaging demonstrated comparable diagnostic performance across most parameters, with VR showing a statistically significant advantage for diagnosis referenced against pathology and trends toward higher accuracy in other domains. In contrast, localization and mediastinal shift assessments yielded similar results between methods. These findings are consistent with prior work demonstrating the limitations of conventional low-dose CT in neonatal thoracic imaging, where overlap between entities such as CPAM and pulmonary sequestration complicates interpretation [4], [5], [6], [7]. The ability to rotate, segment, and selectively visualize anatomical structures in VR provided enhanced clarity, a benefit already highlighted in adult thoracic and liver surgery planning studies [8], [12], [13].

### 3.2 Surgical planning and precision medicine

A key finding was the significant difference in surgical recommendations, with VR assessments more frequently leading to lobectomy compared to conventional CT. This observation mirrors the results of Bakhuis et al., who reported that VR-based planning modified surgical strategy in over 50 % of adult lung segmentectomies [8]. Although our study was not designed to evaluate clinical outcomes, the VR group demonstrated higher accuracy in diagnosis prediction. This underscores VR's potential to improve individualized surgical strategies. This aligns with the paradigm of precision medicine, where interventions are tailored to the patient’s unique anatomical features rather than standardized approaches [1], [2], [3].

An important secondary outcome was the benefit to less experienced surgeons. Junior participants demonstrated improved anatomical recognition and decision-making when supported by VR. This observation resonates with prior research emphasizing that VR and extended reality can mitigate generational gaps in clinical training and usability [14], [15], [16]. By functioning as a health digital twin, VR not only aids decision-making but may also serve as a cognitive equalizer within surgical teams.

In two representative cases, VR visualization revealed that the disease extended beyond the segmental plane, which was not apparent on 2D CT. This prompted a change from segmentectomy to lobectomy, as confirmed by the surgical report. These cases demonstrate VR planning's potential to refine surgical strategies.

### 3.3 Integration into smart hospital ecosystems

Although VR evaluations required longer interpretation times, this was likely influenced by the novelty of the technology and a learning curve effect. Previous studies of immersive planning have similarly noted increased preparation times but emphasized the value of improved intraoperative clarity [9], [11], [12]. Within the broader smart hospital framework, VR-based digital twins can be readily integrated into clinical workflows. PACS-based data export, automated AI segmentation, and commercially available VR platforms [9] make the technology accessible without significant infrastructural changes.

Furthermore, anatomical twins assessed in immersive VR hold promise beyond preoperative planning. Interdisciplinary tumor boards could benefit from shared 3D models to improve communication across specialties. Patient and family counseling may be enhanced by immersive visualization, improving understanding of disease and planned interventions. Surgical training also represents a natural extension, with VR allowing residents to engage in repeated, case-specific practice in a safe environment [10], [11].

The presented framework could potentially integrate with robotic and navigated surgical systems, forming the basis for semi-automated precision surgery pipelines. The display of additional image sources as picture-in-picture visualizations is already possible in the surgeon consoles of robotic systems. Integrating VR content for anatomical orientation during the procedure could be particularly helpful and should be investigated in future studies.

### 3.4 Limitations and future directions

This study is limited by its retrospective, single-center design, thus potential selection bias cannot be excluded. The cohort size (n = 14) reflects the rarity of CTM and limited availability of neonatal low-dose CT data. A formal power analysis was infeasible; instead, the sample represents the full consecutive series meeting inclusion criteria during the study period. Outcomes such as intraoperative accuracy, complication rates, recovery times, and long-term follow-up were not assessed. Future prospective multicenter studies are warranted to validate these results, quantify clinical benefits, and explore the economic implications of VR integration. The study demonstrates an anatomical digital twin, rather than a predictive or biophysical one. Moreover, extending the concept of digital twins beyond static anatomical reconstructions toward functional and predictive models—such as simulating postoperative lung growth and ventilation capacity—represents an important and exciting direction for precision pediatric surgery [11], [15]. In the upcoming future a multimodal integration of detailed anatomical und biophysical data is expected which can also facilitate the integration of haptic features into patient specific simulation and training scenarios.

## 4 Conclusions

Congenital thoracic malformations are complex conditions that challenge preoperative evaluation due to the limitations of low-dose CT imaging in infants [4], [5], [6], [7]. This study shows that VR-based reconstructions can function as health digital twins, providing individualized, interactive models of patient anatomy that improve spatial understanding and influence surgical planning.

Our findings demonstrate non-inferiority of VR compared to conventional CT in diagnostic accuracy, while revealing added benefits in detecting multilobar involvement and mediastinal shifts. VR may alter surgical strategy, consistent with previous reports on the impact of immersive 3D planning in thoracic and cardiac surgery [8], [9], [10], [11], [12]. Importantly, less experienced surgeons could derive particular benefit from VR, suggesting that digital twins can mitigate differences in training level and support more equitable, team-based decision-making [14], [15], [16]. Higher inter-rater agreement in the VR condition suggests that the 3D environment reduces interpretive variability by standardizing spatial understanding, which may improve diagnostic consistency in complex anatomical cases.

Within the smart hospital ecosystem, VR-based anatomical twins are well-positioned to enhance precision and personalized medicine by providing patient-specific roadmaps for surgical care, strengthening interdisciplinary collaboration, and supporting education and counseling. Despite longer evaluation times, the benefits in clarity and decision support justify their integration into pediatric thoracic surgical workflows.

Future research should build on these results through prospective multicenter trials. These studies should correlate findings with intraoperative and long-term outcomes. Another important direction is the exploration of predictive digital twin models to simulate functional dynamics and postoperative trajectories. [11], [13]. By bridging imaging, surgical planning, and precision medicine, VR-based digital twins represent a promising step toward truly individualized care in pediatric thoracic surgery.

## Funding statement

The study was partially financed by the Ministry of Economic Affairs, Industry, Climate Action, and Energy of the State of North Rhine-Westphalia (Project: Innovative Secure Medical Campus).

## Ethics

This study was approved by the Medical Ethical Committee of the University of Bonn (No. 2024-316-BO).

## Declaration of Competing Interest

Authors J.A. and P.F. declare that they hold a minority interest in Medicalholodeck AG. They have also received travel support from Medtronic Germany GmbH, Medicalholodeck AG, and Distalmotion SA. J.A. has received speaker honoraria from Medicalholodeck AG and Chiesi GmbH. Both J.A. and P.F. have received advisory fees from Richard Wolf GmbH. In addition, J.A. and P.F. hold interests in Aesthetic Vision Inc.

All other authors declare that they have no conflicts of interest relevant to this work.
